# 

**Published:** 1913-07

**Authors:** 


### CONTENTS.

ORIGINAL COMMUNICATIONS—

Foreign Bodies in Trachea and Bronchial Tubes. By F. G. Hodgson, M. D., Atlanta, Ga. ............ 146

Imperfect Development a Factor in Genesis of Diseases of Women. By B. S. Moore, M. D., Atlanta, Ga. ............ 148

The Casues of Acquired Insanity. By J. Cheston King, A. B., M. D., Atlanta, Ga. ............ 156

Heredity. By J. W. Williams, M. D., Richmond, Va. . 163

EDITORIAL ............ 170

SELECTIONS AND ABSTRACTS ............ 171

